# Meeting of the Erie County Medical Society

**Published:** 1869-06

**Authors:** 


### Meeting of the Erie County Medical Society.

The Semi-Annual Meeting of this Society was held the second Tuesday in June, and was quite largely attended. The paper read by Dr. Chace will be found in the Original Department of this Journal. No other medical papers were read, and no very important business was brought before the meeting. Drs. John J. Burke, Henry S. Ellwood and A. W. Williams of Buffalo, and Dr. Loren F. Boies of Griffin's Mills, were elected to membership.

A committee was appointed to make arrangements for a social festival to be held in connection with the next annual meeting. This is a proposition which we most heartily approve, and we hope every member of the Society will be present and try to cultivate as much as possible the kindly relations which should exist between the members of the medical profession.

